# Surgical Resection Versus Stereotactic Body Radiation Therapy for Stage I NSCLC: Can Randomized Trials Provide the Solution?

**DOI:** 10.3390/cancers10090310

**Published:** 2018-09-04

**Authors:** Melanie P. Subramanian, Bryan F. Meyers

**Affiliations:** Division of Cardiothoracic Surgery, Washington University School of Medicine, 660 S Euclid Ave, St. Louis, MO 63110, USA; m.p.subramanian@email.wustl.edu

**Keywords:** randomized controlled trials, SBRT, lobectomy, sublobar resection, NSCLC

## Abstract

Surgical resection has traditionally been considered the standard of care for patients with stage I non-small cell lung cancer (NSCLC). With the introduction of stereotactic radiation body therapy (SBRT), there is now a viable option for medically inoperable patients with stage I NSCLC. The effectiveness of SBRT in patients with stage I disease but at elevated surgical risk is unknown. Multiple randomized controlled trials (RCTs) have been attempted to compare surgical resection and SBRT in this population, but have been aborted due to poor patient enrollment. Despite these failures, there still remains a push for more RCTs. In this commentary, we review the challenges that RCTs face in their ability to appropriately compare these two therapies.

## 1. Introduction

Surgical resection has long been considered the standard of care for early-stage lung cancer, with lobectomy representing the optimal resection for most patients. This conclusion came from the landmark 1995 randomized trial published by the Lung Cancer Study Group, which compared lobectomy to sublobar resection for patients treated from 1982–1988 with clinical stage Ia non-small cell lung cancer (NSCLC) [1]. Sublobar resection, which is often less technically challenging, has been considered for patients with smaller tumors, greater comorbidity burden, and presumably less tolerance for loss of an entire pulmonary lobe [2,3,4]. This legacy trial, which enrolled 273 patients, found similar long-term overall survival between the treatment arms of lobectomy and sublobar resection, but observed significantly higher locoregional recurrence rates in patients who received sublobar resection [1]. This prompted the Lung Cancer Study Group to conclude that lobectomy, when feasible, was a standard of care. That conclusion will be challenged or supported in the near future by the trial known as Cancer And Leukemia Group B (CALGB) 140503, which has enrolled far more patients with smaller (≤2 cm) lesions [5]. This cohort will be a more pure collection of true Stage I patients with the use of preoperative FDG-PET screening that was unavailable during the era of the Lung Cancer Study Group trial. While the question of the extent of surgery may lend itself readily to such randomized clinical trials, there are other questions in this Stage I lung cancer realm that are equally important and less easily solved with a trial.

The more recent debate on the management of early-stage lung cancer has come from the introduction and continuous improvement of hypofractionated stereotactic body radiation therapy (SBRT) or stereotactic ablative radiotherapy (SABR). Not all patients with early stage NSCLC will be candidates for even limited surgical resection due to age, comorbid disease, or perceived risks for surgery-related morbidity and mortality. SBRT involves the delivery of high-dose radiation with a high degree of accuracy and precision over a small number of treatment sessions to reduce potential damage to surrounding normal tissue [6]. Over the past decade, SBRT has experienced dramatic increases in utilization in patients deemed medically inoperable or in operable patients electively choosing to avoid surgery. The use of SBRT increased from 6.7% to 16.3% from 2008 to 2013, with a corresponding decrease in lobectomy/pneumonectomy (49.5% to 43.7%) [6]. The rates of wedge resection, conventional radiotherapy, and no treatment remained relatively constant [7]. Compared to standard fractionation radiation therapy, SBRT has demonstrated excellent local control rates. Five-year local control rates associated with standard fractionation therapy have ranged from 30-50%, but have consistently been reported as greater than 90% with SBRT [8,9]. Additionally, given the smaller target volume of lung tissue exposed, there is a much lower toxicity profile associated with SBRT [6,10,11,12]. However, the efficacy of SBRT for use in medically operable patients is still unknown and is the focus of much discussion and debate.

## 2. Comparison of SBRT and Surgical Resection with Randomized Controlled Trials

Many randomized controlled trials (RCTs) have been initiated with the aim of providing high-quality evidence to guide the selection of SBRT versus surgical resection for stage I lung cancer. However, these trials regrettably closed prematurely due to low accrual of patients. The American College of Surgeons Oncology Group Z4099/Radiation Therapy Oncology Group 1021 (ACOSOG 4099/RTOG 1021) trial aimed to compare sublobar resection to SBRT in high-risk, medically operable patients [13]. The trial focused on sublobar resection given (1) higher rates of local recurrence compared to lobectomy and (2) the knowledge that patients undergoing sublobar resection have a comorbidity profile that is comparable to that of patients being considered for SBRT. While the trial aimed to enroll 420 patients to answer the primary question, the study closed after enrolling only 10% of its goal. This disappointing adoption of the trial is despite admirable efforts from study leadership to raise awareness of the trial and alter enrollment criteria to sidestep perceived barriers to enrollment. The so-called “Trial of Either Surgery or Stereotactic Radiotherapy for Early Stage (IA) Lung Cancer” (ROSEL) was a designed as a non-inferiority study to compare SBRT to anatomical surgical resection in medically operable patients [14]. This study did not attempt to focus on high-risk patients and aimed to accrue 960 patients, but closed after 5 years of effort led to enrollment of only 22 patients from 10 sites. Another unsuccessful trial was the “International Randomized Study to Compare CyberKnife Stereotactic Radiotherapy with Surgical Resection in Stage I Non-small Cell Lung Cancer” (STARS), which aimed to compare surgical resection with SBRT in non-high-risk medically operable patients [15]. With a goal accrual of 1030 patients, the trial ended after enrolling 36 patients over an 8-year time span. The common theme of the majority of these trials was the inability to convince stage I lung cancer patients and their physicians to submit to randomization between two mainstream and accepted modes of therapy.

Even though these trials were considered failures, one meta-analysis published by Chang and colleagues attempted to draw conclusions from the pooled data resulting from 58 randomized patients in the ROSEL and STARS trials [10]. The limitations of such an undertaking were substantial: the data were from two different studies, the cohorts were unbalanced, there were extremely few deaths/events, there was <5% of the target accrual deemed suitable at the outset of the trial, and there was short and variable patient follow-up. The authors declared that SBRT could be a viable option based on those data. This conclusion was based on an apparently higher 3-year overall survival among SBRT patients, with similar recurrence free survival between the two arms [16]. However, significant caution is warranted in accepting this conclusion, especially given the small sample size and the risk of a Type I error of inference. Samson and our research group looked at the influence of clinical trial sample size and outcome stability in this exact clinical arena. The outcomes suggest that sample sizes of 100 in the face of three years survival estimates of 70–80% and very heterogeneous enrollment criteria can lead to unstable results of questionable suitability for clinical decision-making [17]. Samson showed that when small clinical trials are simulated with bootstrapping with data from actual clinical practice, perioperative mortality can vary from 0 to 15%, and 3-year overall survival can fluctuate between 46 and 100%; obviously too much outcome variability to accept as clinical guidance. Despite the inherent flaws in their approach and analysis, the meta-analysis authors advocated for more randomized trials to bring clarity to the debate. Currently, there are three additional multicenter randomized trials (STABLE-MATES, VALOR, and POSTILV) underway to compare surgical resection and SBRT [18,19,20,21]. The progress of these studies has not been published. An additional UK-based randomized trial that aimed to compare SBRT and surgical resection in high-risk patients with peripheral tumors (known as SABRTooth) recently closed study recruitment. The results have yet to be published.

## 3. Challenges Faced by RCTs

Why have existing trials failed to adequately recruit sufficient numbers of patients? Enrolling stage I lung cancer patients into a randomized trial when there are two distinct treatment teams may prevent clinicians from suggesting patients for randomization, with the knowledge that their patients may get randomized to the alternate arm [22]. This is in sharp contrast to randomization within a treatment modality, such as a comparison of lobectomy versus sublobar resection or a trial of 48 Gy in 4 fractions versus 60 Gy in 15 fractions. Current trials, including VALOR, are attempting to reduce bias by having study recruitment be performed by more neutral parties (i.e., a pulmonologist with clinical research nurse) or multidisciplinary teams representative of both treatment arms [10]. While many clinical trial leaders and clinicians may have true equipoise between these two treatments in the general sense, this may not reflect the individual patient perspective. For many patients, undergoing a randomization between the disparate options of surgery vs. SBRT may be too much of a leap of faith to allow them to comfortably undergo randomization. Existing RCTs are attempting to address this concern. The STABLE-MATES Trial allows the patients to know in advance the arm to which they have been randomized. They can accept that therapy and be considered randomized, or they can choose the other therapy to which they were not randomized and remain in the study as an observational cohort. While this strategy may improve overall patient recruitment, this approach may raise questions about the ability to generalize the result. There are additional challenges that RCTs face that may prevent them from being effective methods for comparing surgery and SBRT.

One failure of the randomized trial approach to comparing these therapies is that they often fail to focus on a study population where true equipoise exists. For patients with advanced age or formidable comorbidity burden, there seems to be growing general consensus that patients should be referred for SBRT. Likewise, for a patient who is deemed fit to undergo lobectomy, most surgeons would agree that high-quality surgery should be the course of action. The gray area exists where the patient is labeled “high-risk”, a term that has been surprisingly hard to adequately define in most RCTs. Puri and colleagues attempted to look at post-resection outcomes in routine clinical practice for clinical stage IA patients deemed “high-risk” by American College of Surgeons Oncology Group (ACOSOG) criteria (which includes pulmonary function tests, age, and cardiopulmonary function) [23]. Examining 1066 patients who underwent surgery in routine clinical practice, they found that 194 patients (18%) met “high-risk” criteria that had been defined for enrollment in multiple ACOSOG clinical trials. Compared to normal-risk patients, this group of “high-risk” patients were older but had no substantial difference in the overall prevalence of comorbidities including hypertension, coronary artery disease, and diabetes. Despite being labeled “high-risk”, roughly 60% of these patients underwent a lobectomy operation. Among all patients who underwent a lobectomy, major morbidity and hospital mortality were identical between the so-called high-risk and the normal-risk patients. Despite the fact that such a “high-risk” classification is widely used as a part of inclusion-exclusion criteria of some trials, it is likely not representative of true surgical risk from the clinician perspective. Patients’ risk status may be a collection of tangible and intangible factors, including age, functional status, and patient motivation/compliance. These subtle risk factors are nearly impossible to capture in enrollment criteria for traditional RCTs: they are numerous, complex and do not result in an objective “result” that can be used as a source document in a trial enrollment. However, there have been some attempts to broaden the categorization of “high-risk” in existing trials. The SABRtooth trial, which aimed to randomize patients considered high risk for surgical complications into surgical resection or SBRT, did not have strict guidelines to determine whether or not patients are high-risk [24]. In fact, they relied on a multidisciplinary recruitment team to classify whether or not a potentially eligible patient is truly high risk. While they provided guidelines for risk stratification based on pulmonary function, functional status, and established surgical risk classification metrics, the multidisciplinary team had the latitude to consider additional patient factors in their decision to label patients “high-risk”. This approach, however, appears to be the exception and not the rule for RCTs on this subject. The imprecision and subjectivity of risk description can lead to imbalance of treatment cohorts in small to medium sized trials.

Traditional RCT enrollment criteria also do not adequately address the significant heterogeneity in patient risk or tumor characteristics. For example, if a patient with a 5 cm tumor, a Karnofsky performance status of 70, and an FEV1 and DLCO of 40% predicted was screened, that patient would be eligible for the VALOR trial. Similarly, a patient with a 1 cm tumor, KPS of 100 and FEV1/DLCO of 100% predicted would also be a candidate. These two hypothetical patients are markedly different and have vastly different prospects for survival and short-term outcomes. However, they are both eligible for randomization in the same trial simply by strength of the diagnosis of Stage I lung cancer. Stage I disease is heterogeneous, and factors including tumor size, location, and adjacent structures can influence the complexity and outcomes associated with different therapies. For example, a 3.5 cm peripherally located upper lobe tumor is not as challenging to surgically resect when compared to a 1 cm central lesion with close proximity to the bronchus. Factors like tumor location and proximity to adjacent structures also influence variations in SBRT delivered. The American Society for Radiation Oncology (ASTRO) guidelines recommend that patients with centrally located tumors, tumors located near mediastinal structures, or tumors touching or invading the chest wall are at higher risk for adverse events/toxicity, making adjustments to the grade and frequency of administered radiation doses necessary [25]. While these tumor-related characteristics make treatment selection less straightforward, these prognostic factors have not been incorporated in RCT enrollment criteria to study the surgery versus SBRT question. It is interesting to note that the previously mentioned Lung Cancer Study Group trial from 1995 was limited to patients with tumors less than three centimeters and the CALGB 140503 update on that trial has an upper limit of 2 cm tumor size. In surprising contrast, the currently enrolling VALOR trial permits tumors as large as 5 cm in diameter! The challenge of enrollment to clinical trials leads to loosening of enrollment criteria to fill the trial. The resulting loose criteria allow a heterogeneity of trial participants that makes subsequent inference to future patient care very challenging.

## 4. Alternative Study Designs

Given these huge challenges in the execution and subsequent interpretation of the data from RCTs, what are the alternative options? Previous studies using retrospective data from routine clinical practice have attempted to recreate populations with clinical equipoise. Specifically, numerous studies have attempted to match patient and tumor-related characteristics using propensity scores or case matching within large databases. Propensity score methods attempt to address treatment selection bias inherent in observational research. The propensity score itself represents the probability of being assigned to a treatment conditional on observed baseline characteristics [26]. Logistic regression is one commonly used technique to create propensity scores, with the treatment assignment as the dependent variable and all clinically relevant covariates as independent variables. These covariates should include variables that are likely to influence treatment assignment as well as prognosis. Once propensity scores are constructed, multiple techniques can be used to match patients on these scores. In this sense, propensity score matching identifies patients with similar distributions in baseline characteristics, approximating a randomized controlled trial.

Several previous studies have used propensity score matching to compare SBRT and surgical resection. For example, Shirvani and colleagues used the Surveillance, Epidemiology, and End Results (SEER) database linked to Medicare (SEER-Medicare) to compare overall and cause-specific survival in elderly patients (>65 years) receiving lobectomy, sublobar resection, or SBRT [27]. Using propensity scores to match variables including age, gender, Charleson Comorbidity Index, supplemental oxygen use, need for medical assistance, T stage, and mediastinal sampling, the authors derived 251 matched pairs that had undergone either lobectomy or SBRT. However, their matching technique was limited in that they did not attempt matching on individual comorbidities, pulmonary function, or hospital facility type, which may play an important role in treatment assignment (and are often limitations of the databases themselves). Puri and colleagues used the National Cancer Database (NCDB) to compare 30-day surgical mortality and 3-year overall survival in clinical stage I patients receiving surgical resection or SBRT [28]. They used propensity matching, which yielded 5355 matched pairs. Ultimately, 3-year overall survival was higher in patients receiving surgery (69% vs. 46%). The study also performed a separate analysis comparing sublobar resection to SBRT, which resulted in 4555 matched pairs. To simulate a risk gradient, the authors stratified the matched patients into propensity score quintiles and calculated median overall survival in each of these subgroups. They observed that median OS for surgical patients (from lowest to highest propensity score quintile) was 69.5 months, 51.2 months, 47.3 months, 43.9 months, and 39.3 months, respectively. Median overall survival in the matched SBRT group (from lowest to highest propensity score quintile) was 40.9 months, 39.7 months, 32.9 months, 32.2 months, and 28.9 months respectively. With stratification by quintiles, they observed that higher-risk sublobar resection patients and lower-risk SBRT patients had similar median overall survival. However, adequate characterization of these two subgroups for meaningful extrapolation to real world treatment allocation is challenging given that we are confined to patient- and tumor-related variables available in the NCDB, which does not include individual comorbidities, functional status, or pulmonary function. While propensity score matching remains a powerful tool to address treatment selection bias in observational studies, it is imperative that these studies use stringent matching criteria and engage in proper and transparent reporting of methods used. In a meta-analysis of propensity-score studies comparing SBRT and surgical resection, Chen and colleagues found similar disease-free survival between both therapies [29]. Interestingly, they studied the association between propensity matching caliper distance in 8 studies that reported disease free survival. They found a significant association between 0.1 incremental increases in caliper distance and disease-free survival (HR: 1.37, CI: 1.10–1.71). Additionally, the authors observed relatively low adherence to recommended propensity matching quality standards. Only 5 of 14 studies reported standardized mean differences (SMD), and only one study noted <0.1 SMD in all covariates used for matching. The authors concluded that with a stringent propensity score matching methodology and proper reporting of techniques, meta-analyses of propensity-matched studies could be similar to meta-analyses of RCTs.

While large, retrospective studies using sophisticated statistical techniques like propensity score matching are important tools for comparing SBRT vs. surgery have the inherent weaknesses associated with observational studies, they represent practical and effective alternatives to RCTs. RCTs remain limited in the ability to perform adequate risk stratification. Additionally, clinical trials are also difficult and expensive to run. The organizers inevitably face a compromise with regard to the statistical stability of the results versus the cost and practicality of conducting the trial. ROSEL and STARS trials picked sample sizes of 960 and 1030 respectively to answer the question comparing SBRT versus surgery. The currently open STABLE-MATES trial will only randomize 109 patients to each treatment arm in order to detect a much larger effect size in favor of SBRT (HR: 0.46). However, we are skeptical that such a magnitude in effect will be observed, and it is likely more clinically useful to power a study to detect a more nuanced difference. Additionally, such a small number of patients, especially with the potential for highly heterogeneous tumor and comorbidity profiles, will lead to unstable results. Finally, traditional RCTs lack more granular detail that may influence treatment decisions. These deficiencies of clinical trials unmask a possible opportunity for the creation of a detailed, prospective registry of all clinical stage I NSCLC patients who undergo therapy, and lend nicely to a pragmatic trial. Pragmatic clinical trials attempt to study what is occurring in everyday usual care, and examines the comparative effectiveness of therapies based on how they are currently administered [30]. They do not utilize strict inclusion/exclusion criteria, and thus are less likely to face the same issues of inadequate patient recruitment as traditional RCTs. Additionally, pragmatic trials capture the “big picture” of patient risk as determined by his or her treatment team. A pragmatic trial would have the ability to study how patients with early-stage lung cancer are treated in the real world; this often involves multidisciplinary discussion and collaboration in treatment selection that takes into account nuanced patient details. With prospective data collection associated with a pragmatic trial, one would have the ability to collect granular data on factors that played into the treatment allocation, which no RCT today can adequately provide.

## 5. Conclusions

The goal in this specific arena is not to declare a winner: surgery versus SBRT. The true goal is to prospectively collect data that can ultimately benefit and guide multidisciplinary care and personalized medicine. Ideally, clinicians and patients could benefit from a very large and richly annotated dataset with demographics, comorbidities and tumor specific details. Such a registry could begin to approach the notion of personalized medicine by offering new stage I lung cancer patients outcomes data about patients who are “just like them”. Until such a registry is created, special care should be taken in our interpretation and understanding of existing trial data.
